# Yeast Killer Elements Hold Their Hosts Hostage

**DOI:** 10.1371/journal.pgen.1005139

**Published:** 2015-05-14

**Authors:** Reed B. Wickner, Herman K. Edskes

**Affiliations:** Laboratory of Biochemistry and Genetics, National Institute of Diabetes and Digestive and Kidney Diseases, National Institutes of Health, Bethesda, Maryland, United States of America; Fred Hutchinson Cancer Research Center, UNITED STATES

Cytoplasmic linear double-stranded DNA plasmids (or virus-like elements [VLEs]) encoding a secreted toxin, immunity to the toxin, and their own replication and transcription systems were first discovered in the yeast *Kluyveromyces lactis* by Norio Gunge [1]. The *K*. *lactis* toxin, like similar protein toxins produced by similar cytoplasmic linear DNA plasmids in *Pichia acacia* and *Debaryomyces robertsiae* [2–4], kills cells by its highly specific tRNA-cleaving activity, cutting next to the anticodon of tRNA^Gln^ or tRNA^Glu^ [5].

**Fig 1:**
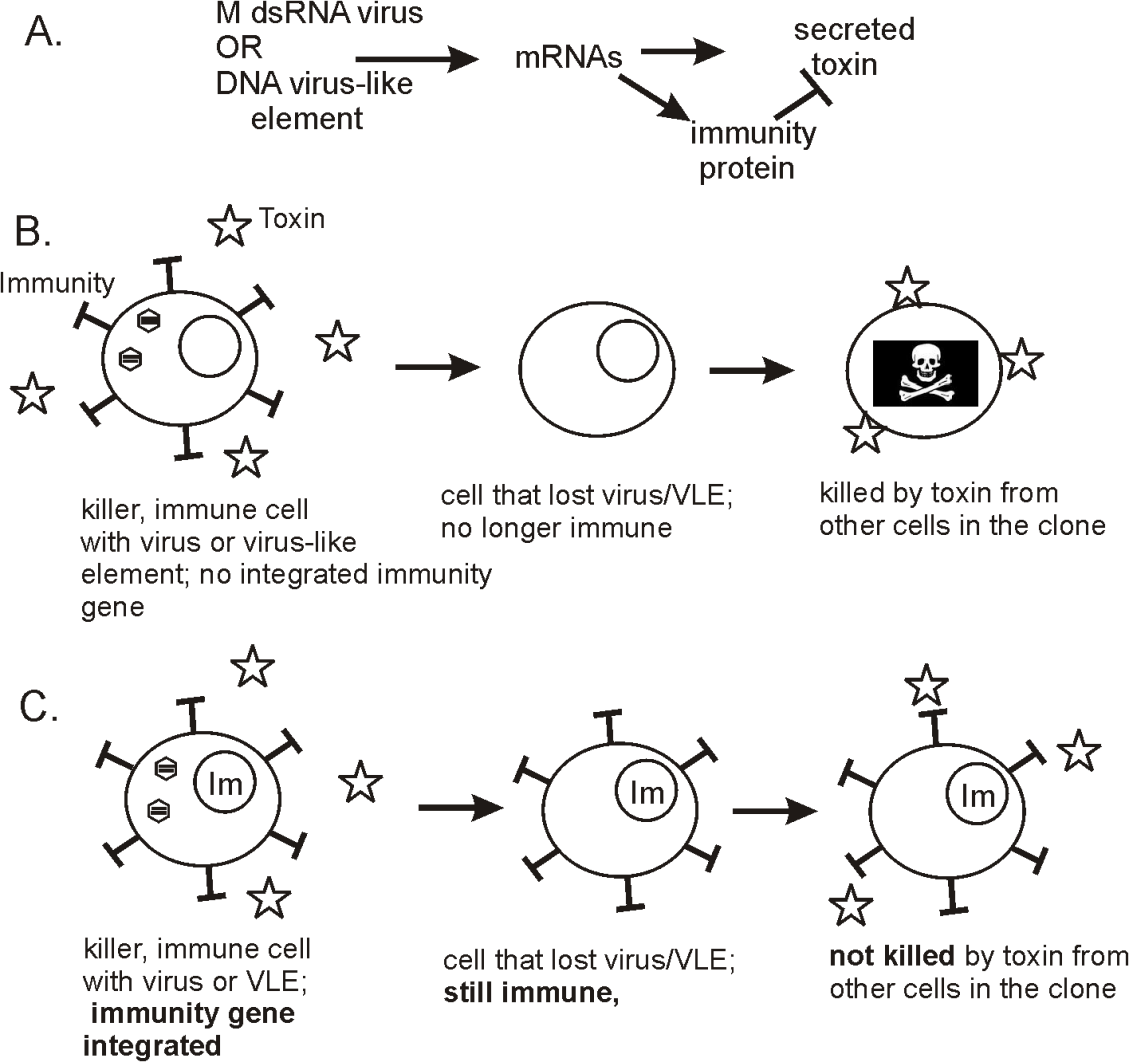
Biology of killer plasmids and viruses of yeasts. (A) Killer elements, whether dsRNA or DNA based, encode a secreted protein toxin and immunity to the toxin. (B) In a clone of killer cells, if one cell loses the virus or virus-like element (VLE), it no longer makes the immunity protein and becomes sensitive to the killer toxin secreted by other cells in the colony. This mechanism enforces maintenance of the killer dsRNA or DNA. (C) If the gene for the immunity protein is integrated into the cell's genome, then loss of the dsRNA virus or DNA plasmid does not result in sensitivity to the toxin, and maintenance of the virus or VLE is no longer enforced.

The immunity protein makes cells insensitive to the corresponding toxin when expressed from one of the cytoplasmic DNA VLEs used as a vector [6] (Fig 1A). However, these immunity proteins are not expressed when their genes are introduced into nuclear circular DNA plasmids under a chromosomal promoter [6]. Now, Meinhardt et al. show that these immunity genes are not expressed because the mRNA is cleaved into multiple small pieces, each polyadenylated [7]. The cytoplasmic DNA VLEs are very A/T rich, and the authors found that the nuclear transcription apparatus recognized several sequences within the immunity gene open reading frame as polyadenylation sites, cleaved the RNA, and appended a polyA 3' tag. To confirm this notion, they recoded the gene, substituting G/C-rich codons for A/T-rich codons, raising the G/C content dramatically. They found that this recoded gene was now well expressed.

The authors interpret their results as a demonstration of a virus-like element mechanism to prevent the cells from incorporating the immunity gene into the nucleus [7]. Such a transfer would make cells able to survive loss of the cytoplasmic VLE because even though the immunity gene on the VLE is lost, the cells are still immune because of the chromosomal copy (Fig 1B and 1C). They state, "From an evolutionary point of view, toxin and immunity functions implemented in VLEs have to be considered as players of an autoselection system rather than providing advantages for the respective host, although the latter clearly benefits from the conferred killer phenotype." This view is particularly interesting and could be tested by determining whether the DNA killer plasmids/VLEs are widespread in wild strains or not. If the killer toxin encoding elements are a net advantage to their hosts, their ability to ignore the rules of meiosis, combined with their weaponizing their host, would make them very common. The mitochondria are a familiar, advantageous non-chromosomal genetic element, which is found in all wild yeasts. If the DNA killer VLEs encoding killer toxins are scarce, it would indicate that they have detrimental effects on the host more than balancing the obvious benefit.

This reasoning may apply as well to the yeast killer systems based on double-stranded RNA in virus particles. There are many such dsRNA-based killer systems, but the most studied system is the L-A virus and its toxin- and immunity-encoding satellite RNA, M dsRNA, in *Saccharomyces cerevisiae*. M dsRNA is encapsidated in and replicated by the Gag major coat protein and Gag-Pol fusion protein, both encoded by L-A dsRNA (reviewed in [8]). The yeast killer toxins are easily detected on plates, but killing is only noticeable when the killer cells far outnumber the sensitive cells. Typically, on plates carefully buffered at the pH optimum for the toxin, about 5 x 10^6^ cells of the toxin-sensitive strain are spread over the entire plate, and a small patch of the killer strain, initially containing perhaps 2 x 10^7^ cells all in one spot, is applied on the lawn. After two days of incubation at 20°C (the optimum temperature for the toxin), the sensitive strain will have formed a lawn except for an area of about 2 mm around the killer strain patch. A killer strain in a small minority among non-killers will not be able to kill other cells.

While the dsRNA-encoded killer toxins provide the host with a weapon, this must not be a very effective weapon. Surveys of wild strains by several groups find only a small minority producing killer toxins. For example, Philliskirk and Young found only 11 killer strains of 592 wild strains of *S*. *cerevisiae* examined [9], while Nakayashiki et al. found no killers among 70 wild strains [10].

Meinhardt et al. have found a mechanism by which the DNA VLEs attempt to enforce their maintenance on the host, killing host cells that have the temerity to withdraw the welcome mat [7]. Most cells in a colony will carry the VLEs, and the few that lose it will be subjected to the action of the toxin (Fig 1B). The implication is that the VLEs were selected to have their very high A/T content (>75%) in a host with a much lower A/T (59%) because that would prevent the host from incorporating the immunity gene into a chromosomal locus, an event that would allow escape from the killer toxin action [7] (Fig 1C).

Drinnenberg et al. found that yeast species that have no RNAi system are just those that have dsRNA virus-based killer systems, and they propose that the advantage of having such armament drove the loss of the RNAi system, which would otherwise eliminate the toxin-encoding dsRNA segment [11]. If the dsRNA toxin-immunity systems have the same autoselection role suggested by Meinhardt et al. for the DNA killer systems then they would not be advantageous on the net, and could not select for loss of the RNAi system. If the killer systems were a net benefit to the host, they would be more widespread. The potential of the toxin-immunity-encoding M segments to damage their hosts is suggested by their conditional or absolute lethality to cells defective in the yeast "innate immunity" system comprising the *SKI* genes [12]. The *SKI* genes block expression of non-polyA mRNAs, such as these viral mRNAs [13]. The mechanism of lethality in this case is completely unknown, and may be as trivial as ineffective immunity to the overproduced toxin. Perhaps the parasitic dsRNA viruses have selected hosts that, for another reason, lack the RNAi system.

In summary, Meinhardt et al. [7] have presented a clear demonstration of a mechanism for a parasite forcing its host to welcome it. Their insightful interpretation of the evolutionary significance of their results may have application beyond the cytoplasmic DNA systems that they studied.
